# Altering the bacterial community: sour soup decreased CO_2_ production and improved the fermentation quality of *Broussonetia papyrifera, Tritriale* and mixed silages

**DOI:** 10.3389/fmicb.2025.1606628

**Published:** 2025-06-25

**Authors:** Qiming Cheng, Dianpeng Liu, Yao Lei, Maoya Li, Yulian Chen, Jiachuhan Wang, Xiangjiang He, Yuanyuan Zhao, Chao Chen, Xiaoqing Zhang

**Affiliations:** ^1^Institute of Grassland Research of Chinese Academy of Agricultural Sciences, Hohhot, China; ^2^College of Animal Science, Guizhou University, Guiyang, China; ^3^Northern Agriculture and Livestock Husbandry Technology Innovation Center, CAAS, Hohhot, China; ^4^Tongliao Institute of Agriculture and Animal Husbandry, Tongliao, China

**Keywords:** sour soup, silage, carbon dioxide, bacterial communities, fermentation

## Abstract

**Introduction:**

Carbon dioxide (CO_2_) generated during the ensiling process is a source of greenhouse gas emissions and a reason for the loss of nutrients during ensiling.

**Methods:**

*Broussonetia papyrifera* (B), *Tritriale* (T) and their mixtures (B7T3, B5T5 and B3T7) were ensiled with sour soup (S) and *Lactobacillus acidophilus* (LAB) to investigate the effects of additives on silage quality, CO2 production and bacterial communities.

**Results:**

After 45 days of fermentation, the B3T7 treatment resulted in the lowest CO_2_ production, a relatively high lactic acid content (pH < 4.2), and relatively high relative abundances of Lactiplantibacillus and Weissella after fermentation; the quality of silage in all the treatments with additives was greater than that in the CK treatment, and the CO_2_ content was significantly lower than that in the CK treatment (*P* < 0.05). In addition, the overall silage quality was better than that of CK after the addition of additives, the CO_2_ content was significantly lower (*P* < 0.05), and adding sour soup resulted in a greater effect than adding LAB. CO_2_ production was positively correlated with the relative abundances of *Lactococcus, Enterococcus*, and *Neoscardovia* and negatively correlated with the relative abundances of *Lactiplantibacillus, Weissella*, and *Sphingomonas*.

**Conclusions:**

In summary, selecting an appropriate proportion of different forages for mixed silage and adding sour soup may be effective ways to improve silage quality and reduce CO_2_ production during ensiling.

## 1 Introduction

Intensification of the greenhouse effect in the atmosphere has led to global warming, precipitating a series of global climate problems that cannot be readily predicted by our current level of scientific understanding. The International Report on the Economics of Climate Change shows that if humans continue to maintain the lifestyles they have today, there is a 50 percent chance that the average global temperature will rise by 4 degrees Celsius by the year 2,100 (Masson-Delmotte et al., 2022). In 2016, many countries worldwide signed the Paris Agreement to combat climate change. China, being among the world's largest emitters of carbon dioxide (CO_2_), has established a goal to curtail CO_2_ emissions by 60–65% relative to the levels in 2005 by the year 2030 (Chen et al., 2019). CO_2_, as one of the principal greenhouse gases, has drawn increasing attention. Approximately 15% of greenhouse gas emissions caused by human activities stem from the livestock industry, according to Adegbeye et al. (2019). Ensiling is a way to preserve the nutritive value of forage over time to not only solve problems related to waste associated with seasonal forage but also provide a source of forage for livestock over the winter (Van Pamel et al., 2011). China produces more than 280 million tons of silage annually, and the global production volume is even greater (Liu et al., 2020). Cai et al. reported that 6 liters of gas can be produced per kg of sorghum silage fermented for 60 days (Cai et al., 1997). Mceniry et al. studied the gases produced during Teagasc ensiling, and they reported CO_2_ levels of up to 90% (McEniry et al., 2011). In recent years, the main concern in silage production has been the quality of the silage, and little attention has been given to CO_2_ emissions during silage production.

*Broussonetia papyrifera* (B) is a tall or shrub-like plant of the genus *Broussonetia* in the family Moraceae. It is widely planted, has a high crude protein content and high biological yield, is rich in nutrients, and contains a variety of bioactive ingredients, which increase the immunity and quality of livestock and poultry and are widely used in animal production (Peng et al., 2019). Although the crude protein content of B leaves is high, their amino acid composition is not balanced, the crude fiber content of the branches and the whole plant is high, the materials are not easy for ruminants to digest when directly fed, and palatability is low; thus, processing is needed to make B an unconventional feed resource with high utilization value (Sun et al., 2022). Common feed processing methods include green hay preparation and silage preparation; however, the biomass of B is high, and the harvest season is typically the rainy season, so green hay is not easy to prepare and preserve (Du et al., 2021). In addition, the high buffer energy value of B, low number of attached lactic acid bacteria, and low water-soluble carbohydrate (WSC) content make traditional ensiling of this material difficult (Cheng et al., 2021). According to previous research reports, the mixing of different forage grasses and the use of silage additives are two effective methods for improving the fermentation quality of silage, and the addition of appropriate silage additives can effectively reduce the production of CO_2_ during the silage process (Ni et al., 2018; Muck et al., 2018). *Triticale* (T) is a new annual grass and forage crop that was formed by the intergeneric hybridization of *Triticum* and *Secale*; it strong disease resistance, wide adaptability, high yield and high nutritional value. After alfalfa (*Medicago sativa* L.) and corn (*Zea mays* L.), it is the industrialized feed crop that is most worthy of promotion and utilization (Nascimento et al., 2015). T has a high WSC content (Li et al., 2023). When B and T are combined for silage, T can provide additional fermentation substrates, promoting silage fermentation, and we predict that a mixed silage consisting of B and T can effectively improve overall silage quality.

Sour soup (S) is traditional food that consists of fermented vegetables, mainly tomato, which is rich in a variety of nutritional and functional ingredients (Lin et al., 2020). At present, the production of S in China has reached 500,000 tons. Due to improper operation, pathogenic bacteria and sulfur-containing compounds are produced during the fermentation process of S, resulting in the waste of one fifth of the S and causing environmental pollution (Lin et al., 2022), whereas ruminants preferred the odor of sour soup due to their unique intestines which are more resistant than those of humans (Thacharodi et al., 2024). S contains many beneficial lactic acid bacteria, including *Lactobacillus namurensis, Pediococcus pentosaceus, Lentilactobacillus buchneri, Lacticaseibacillus casei* and *Lacticaseibacillus rhamnosus* (Xiong et al., 2021). Silage fermentation is driven by lactic acid bacteria under anaerobic conditions, and the dominant genus of S is Lactiplantibacillus. We hypothesized that S could be used as a new type of silage additive to improve silage quality and reduce the production of CO_2_ during the ensiling process. To date, the application of S in silage has not been reported.

The development of sour soup additives for silage applications can result in effectively resource utilization. At present, research on the addition of acid soup to silage is limited. Therefore, the nutritional quality, fermentation quality and microbial community structure of silage with different proportions of B and T were studied and analyzed in this study to reduce the production of CO_2_ and improve the quality of silage.

## 2 Materials and methods

### 2.1 Raw material preparation

The sour soup used in this study is Guizhou red sour soup, primarily composed of fresh cherry tomatoes and small red chili peppers. These ingredients are washed, stemmed, and chopped in specific proportions before being mixed with ginger, garlic, table salt, glutinous rice flour, and distilled liquor. The mixture is then placed in jars and fermented for ~10–20 days in cool, ventilated conditions. The main fermentation products include lactic acid, free amino acids, and functional compounds such as polyphenols and flavonoids (Li, 2022; Liu et al., 2023). The predominant microorganisms involved are lactic acid bacteria and yeast, with key bacterial genera comprising *Lentilactobacillus, Lactiplantibacillus*, and *Levilactobacillus*, while the primary fungal genera are *Kazachstania* and *Pichia* (Liu et al., 2025; Du et al., 2021).

B and T were taken from Jinbi Town, Bijie City, Guizhou Province (25° 73′n, 101° 32′e, Guizhou, China) on June 14, 2022. The study selected the leaf-expansion stage as the harvesting period for *Broussonetia papyrifera*, with the stems and leaves being the primary research focus for cutting. For rye, the harvesting was conducted at the heading stage, with the entire plant being cut. For *Broussonetia papyrifera* (B), dry matter (DM) content accounted for 41.28% of the fresh weight (FW). In terms of nutrient composition, the crude protein (CP) content was 15.56% DM, the neutral detergent fiber (NDF) content was 47.39% DM, the acid detergent fiber (ADF) content was 33.29% DM, and the WSC content was 4.31% DM. The DM content of *Tritriale* (T) constituted 34.21% of the FM. The CP content was 7.25% DM, the NDF content was 57.92% DM, the ADF content was 37.27% DM, and the WSC content was 6.48% DM. B10T0, B7T3, B5T5, B3T7 and B0T1 were obtained by mixing chopped *Broussonetia papyrifera* and *Tritriale* at ratios of 100:0, 70:30, 50:50, 30:70 and 0:100, respectively. Approximately 300 grams of the chopped feed was carefully mixed in a set proportion until a uniform mixture was achieved. Next, the homogeneously mixed feed was carefully hand-packed into polyethylene bags, each measuring 25 cm in width and 30 cm in length. Following this step, a vacuum packaging machine (model SJ-400, manufactured by Shanghai Precision Machinery Manufacturing Co., Ltd.) was employed to remove the air from the bags and seal them. The experimental treatments were established as follows. (1) Control treatment (CK): In this case, 6 milliliters per kilogram of FW of pure distilled water, devoid of any additives, was utilized. (2) Treatment: This treatment included the application of 6 milliliters per kilogram FW of sour soup. (3) LAB treatment: This treatment included the addition of 1 × 10^6^ colony-forming units per gram FW of high-quality *Lactobacillus acidophilus*, sourced from the Zhongke Jiayi Bioengineering Co., Ltd., in Shandong, China. To guarantee the reliability and reproducibility of the experimental findings, three replicates were established for each of these treatments. The total gas (TG) and CO_2_ production were measured after 45 days of ensiling at room temperature (20–25°C), and the relevant indices were determined by opening the bags.

### 2.2 Gas production analysis

Gas production from silage was determined using the drainage method reported by Zheng et al. (2022). Before and after the ensiling process, the silage bags were carefully positioned inside a 5 L beaker. The volume of these bags was precisely measured in a constant-temperature water bath maintained at 25°C. The difference in the bag volume observed before and after ensiling served as an indicator of gas production. To determine the concentration of CO_2_, gas chromatography was employed (Shimadzu GC-20A).

### 2.3 Chemical composition analysis

After 45 days of ensiling, open the silage bags, take ~200 grams of sample from each bag, and dry it in a 105°C oven until a constant weight is achieved to determine the DM content. The dried sample was ground and passed through a 1 mm screen for the analyses of other indices. The CP content was determined via the Dumas combustion method (Gerhardt DUMATHERM; AOAC, 1990). The NDF and ADF contents were determined via an Ankom 200 fiber analyzer system (Ankom Technology Corp.; Zhao et al., 2017). The WSC content was determined via the Thrace-non-sulfuric acid method (Arthur Thomas, 1977).

### 2.4 Analysis of fermentation characteristics

After 45 days of silage fermentation, the bags were opened. A 10-gram sample from each bag was mixed with 90 ml of distilled water, stored at 4°C for 24 h and filtered through four layers of cheesecloth for fermentation index tests. The pH was measured with a portable pH meter. The concentrations of lactic acid (LA), acetic acid (AA), propionic acid (PA), and butyric acid (BA) were measured by HPLC (He et al., 2020), and the ammonia nitrogen (AN) concentration was determined via the phenol-hypochlorite reaction method (Li et al., 2019). This process allowed for a full analysis of silage quality after fermentation.

### 2.5 Microbial community analysis

The 3 samples from each silage treatment were evenly mixed together. The total microbiome DNA in the silage was extracted using a QIamp Fast DNA Stool Mini Kit. A preprimer (5′-ACTCCTACGGGAGGCAGCA-3′) and postprimer (5′-GGACTACHVHHHTWTCTAAAT-3′) were used to obtain the highly variable V3-V4 region of the bacterial 16SrRNA gene. The amplified products were recovered and purified, the sequencing library was prepared using Illunina's TruSeq NanoDNA Library Prep Kit, and the original sequences were analyzed according to a procedure proposed by Wang et al. (2018).

### 2.6 Data analysis

To analyze the impacts of the mixing ratio and additives on fermentation quality as well as the chemical composition of the silages, IBM SPSS 25.0 statistical software was used. The results were evaluated through two-way analysis of variance (ANOVA) and Duncan's multiple range test, with the threshold for statistical significance set to *P* < 0.05. Additionally, regression analysis was used to explore the correlation between gas production and the bacterial community. All graphics presented in this article were generated using GraphPad Prism 9 software.

## 3 Results and discussion

### 3.1 Effects of different additives on the chemical composition of silages

The chemical compositions of B and T after ensiling are shown in Table 1. There was a significant effect of the additives, mixing ratios and their interaction on the DM, CP, ADF and WSC contents of the silages (*P* < 0.001). With increasing proportion of B in the silage, the CP content significantly increased (*P* < 0.05), whereas the dry matter (DM), neutral detergent fiber (NDF), and acid detergent fiber (ADF) contents significantly decreased (*P* < 0.05). This may be related mainly to the properties of the material under study. This may have occurred because the T samples selected for this study were harvested at the late tasseling stage, and the fresh T samples presented relatively high NDF (57.92% DM) and ADF (37.27% DM) contents, whereas the fresh B samples presented relatively high CP (15.56% DM) contents. This is in line with the results of Ni et al. (2018), who reported that with increasing amounts of raw materials with high CP and fiber contents, the CP and fiber contents of mixed silage increases. The WSC content of all the treatments decreased with decreasing proportion of B (*P* < 0.05). This may have occurred due to the high content of antimicrobial substances such as polyphenols and flavonoids in B, which can inhibit microbial activity and reduce microbial utilization of WSCs (He et al., 2020). At the same ratio, the contents of DM, CP and WSCs were greater in the S treatment than in the LAB and CK treatments. This may have occurred due to the high content of lactic acid bacteria and organic acids in sour soup, which can facilitate the rapid formation of an acidic environment and inhibit the utilization of DM, CP and WSCs by undesirable microorganisms (Li et al., 2021). At the same ratio of silage materials, the NDF and ADF contents in the S treatment were lower than those in the LAB and CK treatments. Zhao et al. (2018) reported that *Lactiplantibacillus plantarum* can reduce the fiber content of silage. Additionally, Liao et al. (2022) reported that Saccharomyces cerevisiae and *Lactobacillus brevis* can reduce the fiber content of silage. Sour soup contains many microorganisms (lactic acid bacteria and yeasts), which may lead to a reduction in the NDF and ADF content of silage due to the ability of certain yeasts and lactic acid bacteria to degrade cellulose (Lin et al., 2020). The above results revealed that the mixing ratio of silage materials significantly affected the chemical composition of the silage and that the addition of sour soup effectively preserved nutrients and reduced the fiber content of the silage.

**Table 1:** Analysis of the chemical composition of mixed B and T silage with different additives.

**Items**	**Treatment (T)**	**Mixing proportion (M)**					**Mean^T^**	**SEM**	***P*** **value**		
		**B10T0**	**B7T3**	**B5T5**	**B3T7**	**B0T10**			**T**	**M**	**T × M**
DM %FM	CK	27.37^Db^	29.17^Cb^	33.19^Bab^	34.92^B^	39.50^A^	32.83	0.001	0.02	< 0.001	0.004
	S	30.26^Ea^	32.34^Da^	34.06^Ca^	35.93^B^	38.12^A^	34.14				
	LAB	29.19^Dab^	30.15^Db^	32.19^Cb^	35.31^B^	39.59^A^	33.29				
	Mean^M^	28.94	30.55	33.15	35.39	39.07					
CP %DM	CK	13.66^Aa^	11.46^Bb^	7.71^Cc^	6.32^Dc^	5.52^Ec^	9.02	0.062	< 0.001	< 0.001	< 0.001
	S	14.71^Aa^	13.66^Ba^	12.73^Ca^	10.66^Da^	8.25^Ea^	12.00				
	LAB	14.32^Ab^	11.89^Bb^	9.18^Cb^	8.62^Cb^	7.03^Db^	10.08				
	Mean^M^	14.23	12.03	9.19	8.53	6.93					
NDF %DM	CK	46.83^E^	53.33^D^	56.81^C^	60.18^Ba^	65.59^Aa^	56.55	0.001	0.139	< 0.001	0.014
	S	44.51^E^	50.81^D^	53.80^C^	55.64	60.40	53.03				
	LAB	46.05^E^	51.42^D^	55.21^C^	58.04^Bab^	63.61^Aab^	55.66				
	Mean^M^	46.86	51.29	54.56	57.85	65.23					
ADF %DM	CK	35.16^Ea^	37.60^Da^	40.18^Ca^	43.17^Ba^	46.53^Ab^	40.53	0.001	0.006	< 0.001	0.006
	S	33.40^D^	36.02^CD^	39.99^B^	41.17^BCb^	44.01^Ab^	38.92				
	LAB	34.59^Db^	37.42^Ca^	42.98^Bb^	43.61^Bb^	45.66^Aa^	40.85				
	Mean^M^	34.70	38.74	41.17	39.84	41.04					
WSC %DM	CK	2.19^Ab^	2.14^Aa^	2.13^Ab^	1.85^Bb^	1.48^C^	1.96	0.022	< 0.001	< 0.001	< 0.001
	S	4.20^Aa^	3.23^Ba^	1.90^Cb^	1.57^Db^	1.67^CD^	2.52				
	LAB	3.35^Ac^	3.47^Ab^	1.71^Ba^	1.51^Ba^	1.50^B^	2.31				
	Mean^M^	3.25	2.95	1.91	1.65	1.55					

CK, Control treatment (devoid of any additives); S, included the application of 6 milliliters per kilogram FW of sour soup; LAB, included the addition of 1 × 10^6^ colony-forming units per gram FW of high-quality *Lactobacillus acidophilus*; B10T0, obtained by mixing chopped *Broussonetia papyrifera* and *Tritriale* at ratios of 100:0; B7T3, obtained by mixing chopped *Broussonetia papyrifera* and *Tritriale* at ratios of 70:30; B5T5, obtained by mixing chopped *Broussonetia papyrifera* and *Tritriale* at ratios of 50:50; B3T7, obtained by mixing chopped *Broussonetia papyrifera* and *Tritriale* at ratios of 30:70; B0T10, obtained by mixing chopped *Broussonetia papyrifera* and *Tritriale* at ratios of 0:100; A–E, mixing ratios within a row with different superscripts differ under the same additive treatment (*P* < 0.05); a–c, additive treatment within a column with different superscripts differs at the same mixing ratio (*P* < 0.05); DM, dry matter; CP, crude protein; WSC, water-soluble carbohydrate; NDF, neutral detergent fiber; ADF, acid detergent fiber; T, treatments; M, mixing proportion; T × M, interaction between treatments and mixing proportion; SEM, standard error of the mean.

### 3.2 Effects of different additives on the fermentation quality of silages

As shown in Table 2, the additives, mixing ratios and their interactions had significant effects on the silage pH and fermentation products (*P* < 0.05). The pH of the silage was greater (*P* < 0.05) when the proportion of B was greater. Due to the low WSC content and high buffer energy value of B, the pH of this material is difficult to reduce, even at a high LA content (Si et al., 2018). Numerous studies have shown that forage crops that are difficult to ferment during ensiling, such as legumes and woody crops, exhibit good results in mixed silage. Similar results were obtained by Li et al. (2022) for a mixed silage of broad bean and oats, who reported that the best overall silage quality was obtained from a mixture of 30% broad bean and 70% oats. Similar results were obtained by Zong et al. (2023) for a mixed silage of whole-plant corn and hairy vetch, who reported that the silage quality was better when the proportion of maize was 60–80%. LA is a direct product of DM and WSC fermentation by lactic acid bacteria, which influence the whole silage process and play an important role in determining silage quality and silage preservation time (Ni et al., 2017). The LA content of the B3T7 treatment was significantly greater (*P* < 0.05) than that of the other treatments under the same additives, indicating that silage fermentation is favored under a B to T ratio of 3:7. Keshri et al. (2019) reported that aerobic spoilage was inhibited under an AA content of < 3% DM. The AA content of all the treatments in this study ranged from 0.85 to 2.75% DM, which ensured aerobic stabilization of the silage. The formation of BA is usually undesirable during silage, as it implies nutrient loss and energy depletion (Muck, 2010). BA was not detected in any of the treatments in this study, which implies that the treatments were not subject to secondary fermentation. AN production usually implies CP degradation (Pahlow, 2003). The AN content decreased with decreasing percentage of B. This may have been influenced mainly by the material properties of B and T. Due to the higher CP content of B (15.56% DM) than T, the AN content increased following B degradation. In addition, this may be related to the higher buffer energy value of B, as a result of which B is less likely to successfully ferment during ensiling, nutrients are less likely to be preserved, and protein degradation is likely to occur; these are in line with the findings of Sun et al. (2022). Numerous studies have shown that additives are effective in improving silage quality (Dunière et al., 2013). The same results were observed in the present study, where the S-added treatments had lower pH values; lower AA, PA and AN contents; and higher LA contents than did the CK treatment. This may be related to the presence of organic acids and numerous microorganisms in the sour soup, whose presence led to the rapid formation of an acidic environment, lowering the pH of the silage and inhibiting the activity of undesirable microorganisms (Xiong et al., 2021). These findings suggest that the addition of sour soup can effectively inhibit CP degradation, reduce PA production and lower silage nutrient losses, which is consistent with the observation that the S treatment resulted in a higher CP content, as shown in Table 1. The above results indicated that among the treatments with different mixing ratios, the fermentation quality of the B3T7 treatment was better than that of the other treatments (pH < 4.2, higher LA content, and lower AA, PA and AN contents), and the addition of sour soup effectively improved the fermentation quality of the silage.

**Table 2:** Analysis of silage quality under mixed B and T silage with different additives.

**Items**	**Treatment (T)**	**Mixing proportion (M)**					**Mean^T1^**	**SEM**	***P*** **value**		
		**B10T0**	**B7T3**	**B5T5**	**B3T7**	**B0T10**			**T**	**M**	**T × M**
pH	CK	5.77^Aa^	5.64^Ba^	5.21^Ca^	4.54^Da^	4.21^Ea^	5.07	0.008	0.045	< 0.001	< 0.001
	S	5.48^Ab^	5.20^Bc^	4.52^Cb^	3.91^Eb^	4.12^Db^	4.64				
	LAB	5.61^Aab^	5.41^Bb^	4.54^Cb^	4.04^Eb^	4.15^Db^	4.72				
	Mean^M^	5.62	5.41	4.75	4.19	4.16					
LA %DM	CK	3.22^Db^	3.57^Cb^	4.16^Bb^	4.97^Ab^	3.91^Bb^	3.96	0.038	< 0.001	< 0.001	< 0.001
	S	3.62^Ca^	4.38^Ba^	4.51^Ba^	5.51^Aa^	4.27^Ba^	4.45				
	LAB	3.19^Db^	3.70^Cb^	4.46^Ba^	5.63^Aa^	4.33^Ba^	4.26				
	Mean^M^	3.34	3.88	4.37	5.37	4.17					
AA %DM	CK	2.71^A^	2.39^ABb^	2.34^AB^	2.08^Ba^	0.85^Cb^	2.08	0.034	0.135	< 0.001	< 0.001
	S	2.55^A^	2.14^ABb^	2.04^B^	1.93^Bb^	1.02^Ca^	1.94				
	LAB	2.13^B^	2.20^Aa^	2.08^B^	2.14^Ba^	0.89^Cb^	1.99				
	Mean^M^	2.46	2.58	2.15	2.05	0.92					
PA %DM	CK	2.04^Aab^	2.09^Aa^	1.38^Bb^	1.80^Ba^	-	1.46	0.024	0.251	< 0.001	< 0.001
	S	2.35^Aa^	1.78^Bab^	1.46^Cb^	1.34^Cb^	-	1.38				
	LAB	1.88^Ab^	1.59^Bb^	1.69^Ba^	1.69^Ba^	-	1.37				
	Mean^M^	2.09	1.82	1.51	1.61	-					
BA %DM	CK	-	-	-	-	-		-	-	-	-
	S	-	-	-	-	-					
	LAB	-	-	-	-	-	-				
	Mean^M^	-	-	-	-	-					
AN %DM	CK	2.52^Aa^	2.54^A^	2.20^AB^	2.04^BC^	1.75^C^	2.21	0.035	0.077	< 0.001	0.094
	S	2.12^Ab^	2.00^AB^	2.08^AB^	2.07^AB^	1.62^C^	1.98				
	LAB	2.06^Ab^	2.02^A^	2.05^A^	2.02^A^	1.66^B^	1.96				
	Mean^M^	2.40	2.35	2.14	2.05	1.68					

CK, Control treatment (devoid of any additives); S, included the application of 6 milliliters per kilogram FW of sour soup; LAB, included the addition of 1 × 10^6^ colony-forming units per gram FW of high-quality *Lactobacillus acidophilus*; B10T0, obtained by mixing chopped *Broussonetia papyrifera* and *Tritriale* at ratios of 100:0; B7T3, obtained by mixing chopped *Broussonetia papyrifera* and *Tritriale* at ratios of 70:30; B5T5, obtained by mixing chopped *Broussonetia papyrifera* and *Tritriale* at ratios of 50:50; B3T7, obtained by mixing chopped *Broussonetia papyrifera* and *Tritriale* at ratios of 30:70; B0T10, obtained by mixing chopped *Broussonetia papyrifera* and *Tritriale* at ratios of 0:100; A–E, mixing ratios within a row with different superscripts differ under the same additive treatment (*P* < 0.05); a–c, additive treatment within a column with different superscripts differs at the same mixing ratio (*P* < 0.05); LA, lactic acid; AA, acetic acid; PA, propionic acid; BA, butyric acid; AN, ammonia nitrogen; T, treatments; M, mixing proportion; T × M, interaction between treatments and mixing proportion; SEM, standard error of the mean.

### 3.3 Effects of different additives on the bacterial diversity of silages

The α diversity of the silage samples obtained from each treatment is shown in Table 3. The average coverage of all the samples was >0.998, indicating that the sequencing process was able to adequately represent the bacterial diversity of the silage samples (Yang et al., 2019). The Chao1, Shannon and Simpson indices of the treatments decreased with a decreasing proportion of B, and those of B3T7 were the lowest (*P* < 0.05). In addition, the additives had significant effects on the microbial diversity of the mixed B and T silage (*P* < 0.05). At the same mixing ratio, the Chao1, Shannon and Simpson indices were lowest in the S treatment (*P* < 0.05). These findings indicate that lactic acid bacteria were dominant and that the addition of sour soup reduced the relative abundance of other bacteria and promoted the reproduction of lactic acid bacteria.

**Table 3:** Analysis of α bacterial diversity in silage.

**Items**	**Treatment (T)**	**Mixing proportion (M)**					**Mean^T1^**	**SEM**	***P*** **value**		
		**B10T0**	**B7T3**	**B5T5**	**B3T7**	**B0T10**			**T**	**M**	**T × M**
Chao1	CK	493.62^A^	340.01^B^	368.09^B^	363.63^B^	501.36^A^	413.34	5.396	< 0.001	< 0.001	< 0.001
	S	380.13^A^	305.84^B^	222.00^C^	231.35^AB^	285.80^AB^	285.03				
	LAB	447.77^A^	376.48^AB^	408.07^AB^	255.78^C^	355.27^B^	368.67				
	Mean^M^	440.51	340.78	332.72	283.59	380.81					
Shannon	CK	6.23^A^	6.21^A^	5.72^B^	5.47^B^	5.67^B^	5.86	0.039	0.001	0.002	0.002
	S	6.33^A^	6.10^AB^	5.49^C^	5.73^BC^	6.52^A^	6.03				
	LAB	6.06	6.32	6.27	6.28	6.36	6.26				
	Mean^M^	6.21	6.21	5.82	5.82	6.18					
Simpson	CK	0.973	0.966	0.960	0.948	0.966	0.963	0.001	0.398	< 0.001	0.363
	S	0.976	0.981	0.955	0.955	0.964	0.966				
	LAB	0.968	0.975	0.966	0.954	0.968	0.966				
	Mean^M^	0.973^A^	0.966^AB^	0.960^B^	0.948^C^	0.966^AB^					
Coverage	CK	0.999	0.998	0.998	0.998	0.998	0.998				
	S	0.999	0.999	0.999	0.999	0.999	0.999				
	LAB	0.999	0.999	0.999	0.999	0.999	0.999				
	Mean^M^	0.999	0.999	0.999	0.999	0.999					

CK, Control treatment (devoid of any additives); S, included the application of 6 milliliters per kilogram FW of sour soup; LAB, included the addition of 1 × 10^6^ colony-forming units per gram FW of high-quality *Lactobacillus acidophilus*; B10T0, obtained by mixing chopped *Broussonetia papyrifera* and *Tritriale* at ratios of 100:0; B7T3, obtained by mixing chopped *Broussonetia papyrifera* and *Tritriale* at ratios of 70:30; B5T5, obtained by mixing chopped *Broussonetia papyrifera* and *Tritriale* at ratios of 50:50; B3T7, obtained by mixing chopped *Broussonetia papyrifera* and *Tritriale* at ratios of 30:70; B0T10, obtained by mixing chopped *Broussonetia papyrifera* and *Tritriale* at ratios of 0:100; A–C, mixing ratios within a row with different superscripts differ under the same additive treatment (*P* < 0.05); T, treatments; M, mixing proportion; T × M, interaction between treatments and mixing proportion; SEM, standard error of the mean.

**Figure 1:**
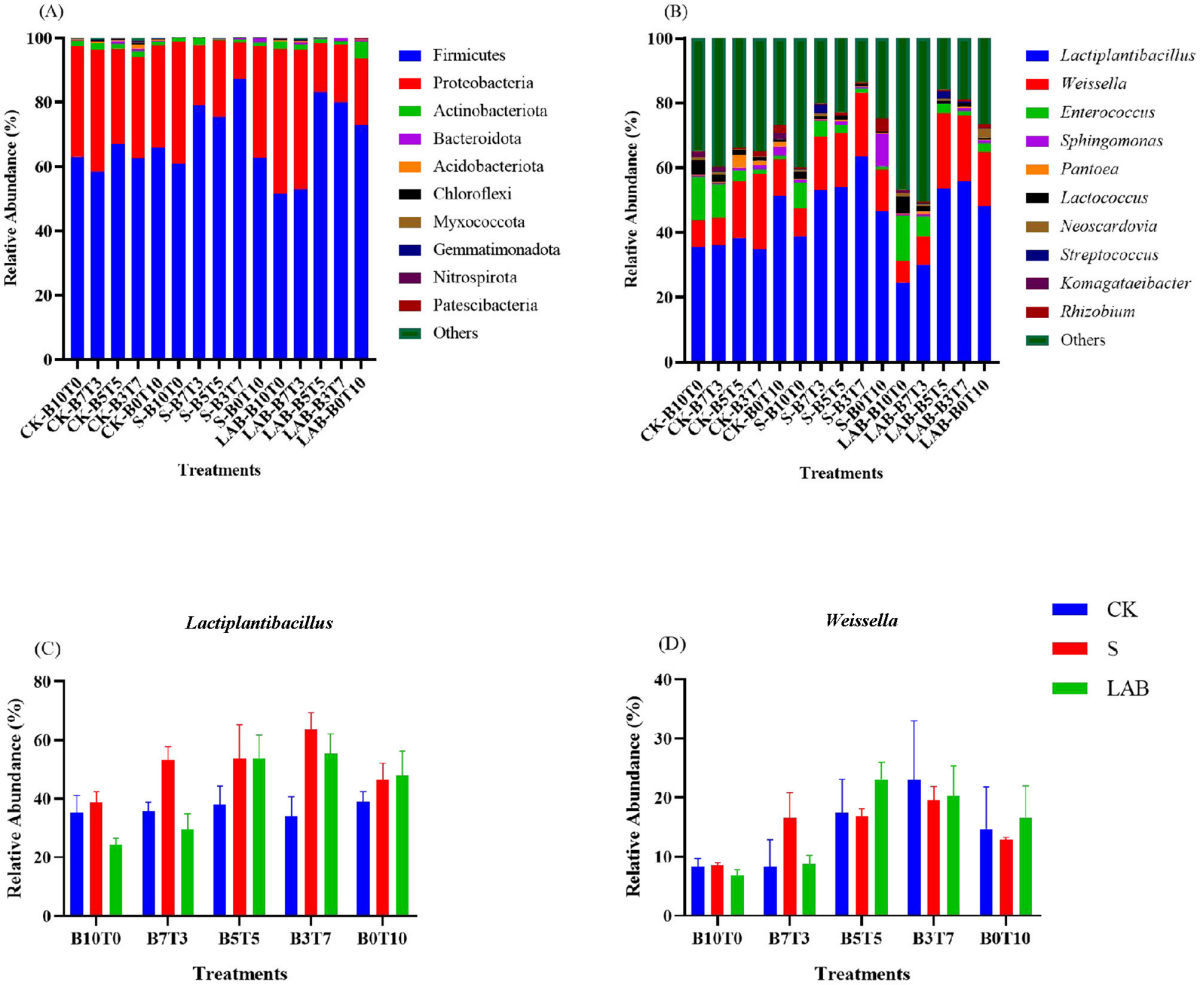
Microbial communities in silages after 60 days of ensiling. Bacterial communities are reported at the phylum level **(A)**, the genus level **(B)** and the relative abundances of *Lactiplantibacillus* **(C)** and *Weissella* **(D)**. CK, Control treatment (devoid of any additives); S, included the application of 6 milliliters per kilogram FW of sour soup; LAB, included the addition of 1 × 10^6^ colony-forming units per gram FW of high-quality *Lactobacillus acidophilus*; B10T0, obtained by mixing chopped *Broussonetia papyrifera* and *Tritriale* at ratios of 100:0; B7T3, obtained by mixing chopped *Broussonetia papyrifera* and *Tritriale* at ratios of 70:30; B5T5, obtained by mixing chopped *Broussonetia papyrifera* and *Tritriale* at ratios of 50:50; B3T7, obtained by mixing chopped *Broussonetia papyrifera* and *Tritriale* at ratios of 30:70; B0T10, obtained by mixing chopped *Broussonetia papyrifera* and *Tritriale* at ratios of 0:100.

The relative abundance of microorganisms at the phylum level is shown in Figure 1A. The phyla Firmicutes and Proteobacteria were the main phyla after 45 days of mixed B and T ensiling, which is in agreement with the results of Wang et al. (2022). In this study, the relative abundance of Firmicutes was lower when the proportion of B was greater. Studies have reported that due to the high buffer energy values of legumes and some woody feeds with high protein contents, the pH of these materials is difficult to reduce, and studies have shown that acidic environments are more suitable for the growth of Firmicutes, so the relative abundance of Firmicutes is low when the proportion of B is high (He et al., 2021). The relative abundance of genus-level microorganisms is shown in Figure 1B. In this study, *Lactiplantibacillus, Weissella* and *Enterococcus* were the main genera in the silage samples. *Enterococci* are silage probiotics that act mainly in the early stages of ensiling, as they do not tolerate acidic environments and are usually replaced by lactic acid bacteria in the later stages of ensiling (Delpech et al., 2019). In this study, a greater abundance of *Enterococcus* was detected when the ratio of B was greater. This may have occurred because an acidic environment was not formed when the ratio of B was high, which allowed *Enterococcus* strains to survive. The presence of *Enterobacter* and *Clostridia* during silage fermentation is considered unfavorable, as these bacteria compete with lactic acid bacteria for fermentation substrates, slowing lactic acid production and increasing protein degradation (Dunière et al., 2013). In this study, the abundances of *Enterobacte*r and *Clostridia* were below the detection level, and BA was not detected (Table 2), which indicated that butyric acid was not formed in the silage. Ensiling is a *Lactobacillus*-driven fermentation process, and *Lactobacilli* gradually take over as the ensiling process progresses (Muck, 2013). *Lactiplantibacillus* are lactic acid-producing microorganisms in the ensiling process, and there is a significant correlation between the abundance of these microorganisms and lactic acid content (Guan et al., 2018). Compared with those in the B silage alone, the relative abundances of *Lactobacilli* (Figure 1C) and *Weissella* (Figure 1D) gradually increased with increasing proportion of T in the silage, and the relative abundances of lactic acid bacteria and *Weissella* in the B3T7 mixture were greater than those in the other treatments, which indicated that the optimum fermentation environment was formed under this proportion. This finding is in line with the results of Wang et al. (2019), who reported that mixed alfalfa and maize silage was best ensiled at ratios of 4:6 and 6:4. Under the same mixing ratio, the addition of sour soup effectively improved the fermentation environment of the silage, and the relative abundance of lactic acid bacteria in the S treatment was greater than that in the CK and LAB treatments, which may be attributed to the various nutrients (organic acids, vitamins, minerals, etc.) and the large number of microorganisms (mainly lactobacilli) that were present in the silage fermentation environment (Lin et al., 2020; Choi et al., 2019). Moreover, this study revealed that the additives did not have a good effect on the ensiling of B and T alone, which may be related to the properties of the materials. B has few lactic acid bacteria attached to the material, a low WSC content, and abundant bioactive compounds, including flavonoids and terpenes with antioxidant and anti-inflammatory properties, which are not conducive to silage fermentation (Chen et al., 2023). Many studies have shown that T, which is rich in fermentation substrates, can ferment quickly without additives and that the effects of additives are not obvious. The above results revealed that the mixing ratio affected the bacterial community composition of the B and T silages, that the optimal B proportion was 30%, and that the addition of sour soup effectively promoted the growth of *Lactiplantibacillus* and *Weissella*.

### 3.4 Effects of different additives on CO_2_ production and TG volume of silages

Silage fermentation is a complex process that generally includes four stages: an aerobic respiration period, an anaerobic microbial competition period, a lactic acid accumulation period and a relatively stable period (Murdoch, 1961). In the aerobic fermentation stage, due to plant cell and aerobic microbial respiration, nutrients are consumed, which results in the production of many gases. The production of these gases not only results in the loss of nutrients but also may cause greenhouse gas emissions (Guo et al., 2021). The effects of different additives on CO_2_ and TG production in the mixed B and T silage are shown in Table 4. The additives and their blending proportions significantly influenced CO_2_ and TG yields in silage (*P* < 0.001). With changing B-to-T ratio, the CO_2_ and TG production exhibited the same trend. The carbon dioxide and TG production tended to decrease and then increased as the proportion of T increased, with the B3T7 treatment resulting in the lowest carbon dioxide content and TG production. This may have occurred because the mixing ratio for B3T7 resulted in a silage environment that facilitated microbial fermentation, and the microorganisms in the additives utilized the substrates to produce more LA, which led to a lower-pH silage environment, inhibited the growth of harmful bacteria, and reduced the nutrient loss, thus lowering gas production. At the same mixing ratio, the LAB treatment significantly (*P* < 0.05) reduced TG production and CO_2_ production compared with those under CK. This may have occurred because the addition of lactic acid bacteria facilities the rapid formation of an acidic environment, inhibiting the aerobic respiration phase of the ensiling and creating a new microbial community structure, which in turn reduces CO_2_ production. This finding is consistent with the findings of Cai et al. (1997), who also showed that the addition of lactic acid bacteria was effective in reducing CO_2_ production during ensiling. In this study, the S treatment reduced TG production and CO_2_ production more effectively than the LAB treatment (*P* < 0.05). There are two reasons why S treatment is more effective than LAB treatment: one is that S itself is more acidic, which is conducive to the rapid formation of an acidic environment, and the other reason is that sour soup contains a mixed flora of various beneficial lactic acid bacteria, whereas the LAB used in this study only consisted of a single strain, which was not as effective as the sour soup. As mentioned above, both the mixing ratio and additives significantly affected CO_2_ production and TG production, and the addition of sour soup to B3T7 resulted in the lowest CO_2_ production and TG production. The primary sources of CO_2_ are plant respiration and microbial metabolic activities, while the reduction of CO_2_ emissions mainly involves either decreasing CO_2_ production or consuming the already produced CO_2_. The results of this study indicate that adding sour soup accelerates the silage fermentation process, promoting the formation of an acidic anaerobic environment, thereby inhibiting plant respiration and microbial metabolic activities. Additionally, mixed silage with different raw materials creates a survival environment for CO_2_-fixing microorganisms, further reducing CO_2_ production. According to the results of this study, under the mixing ratio employed in B3T7, CO_2_ production was reduced by 130.56 ml per 100 g of S-treated silage compared with that in the CK treatment, and CO_2_ production was reduced by 53.97 ml per 100 g of S-treated silage compared with that in the LAB treatment. China's annual silage production volume is ~280 million tons, and the global silage production volume is even greater. Therefore, selecting an optimal proportion of mixed silage consisting of different crops and adding sour soup may be effective ways to reduce CO_2_ emissions during ensiling.

**Table 4:** Analysis of CO_2_ and TG production.

**Items**	**Treatment (T)**	**Mixing proportion (M)**					**Mean^T1^**	**SEM**	***P*** **value**		
		**B10T7**	**B7T3**	**B5T5**	**B3T7**	**B0T10**			**T**	**M**	**T × M**
CO_2_(ml)	CK	549.55^Aa^	400.49^Ba^	361.93^Ca^	333.09^Da^	298.50^Ea^	388.71	3.28	< 0.001	< 0.001	0.305
	S	390.73^Ac^	286.97^Bc^	254.13^Cc^	158.17^Ec^	195.73^Dc^	257.15				
	LAB	445.58^Ab^	344.93^Bb^	304.53^Cb^	210.29^Eb^	250.28^Db^	311.12				
	Mean^M^	461.95	344.13	306.86	233.85	248.17					
TG (ml)	CK	729.36^Aa^	595.75^Ba^	589.75^Ba^	459.74^Da^	511.98^Ca^	577.32	2.04	< 0.001	< 0.001	< 0.001
	S	533.88^Ac^	446.22^Bc^	400.31^Cc^	324.33^Ec^	377.49^Dc^	416.45				
	LAB	603.49^Ab^	522.78^Bb^	457.21^Cb^	375.81^Eb^	443.76^Db^	480.61				
	Mean^M^	622.24	521.58	482.42	386.63	444.41					

TG, total gas; CK, control treatment (devoid of any additives); S, included the application of 6 milliliters per kilogram FW of sour soup; LAB, included the addition of 1 × 10^6^ colony-forming units per gram FW of high-quality *Lactobacillus acidophilus*; B10T0, obtained by mixing chopped *Broussonetia papyrifera* and *Tritriale* at ratios of 100:0; B7T3, obtained by mixing chopped *Broussonetia papyrifera* and *Tritriale* at ratios of 70:30; B5T5, obtained by mixing chopped Broussonetia papyrifera and *Tritriale* at ratios of 50:50; B3T7, obtained by mixing chopped *Broussonetia papyrifera* and *Tritriale* at ratios of 30:70; B0T10, obtained by mixing chopped *Broussonetia papyrifera* and *Tritriale* at ratios of 0:100; A–E, mixing ratios within a row with different superscripts differ under the same additive treatment (*P* < 0.05); a–c, additive treatment within a column with different superscripts differs at the same mixing ratio (*P* < 0.05); T, treatments; M, mixing proportion; T × M, interaction between treatments and mixing proportion; SEM, standard error of the mean.

### 3.5 Analysis of correlations of bacterial relative abundance with fermentation products and gas production

**Figure 2:**
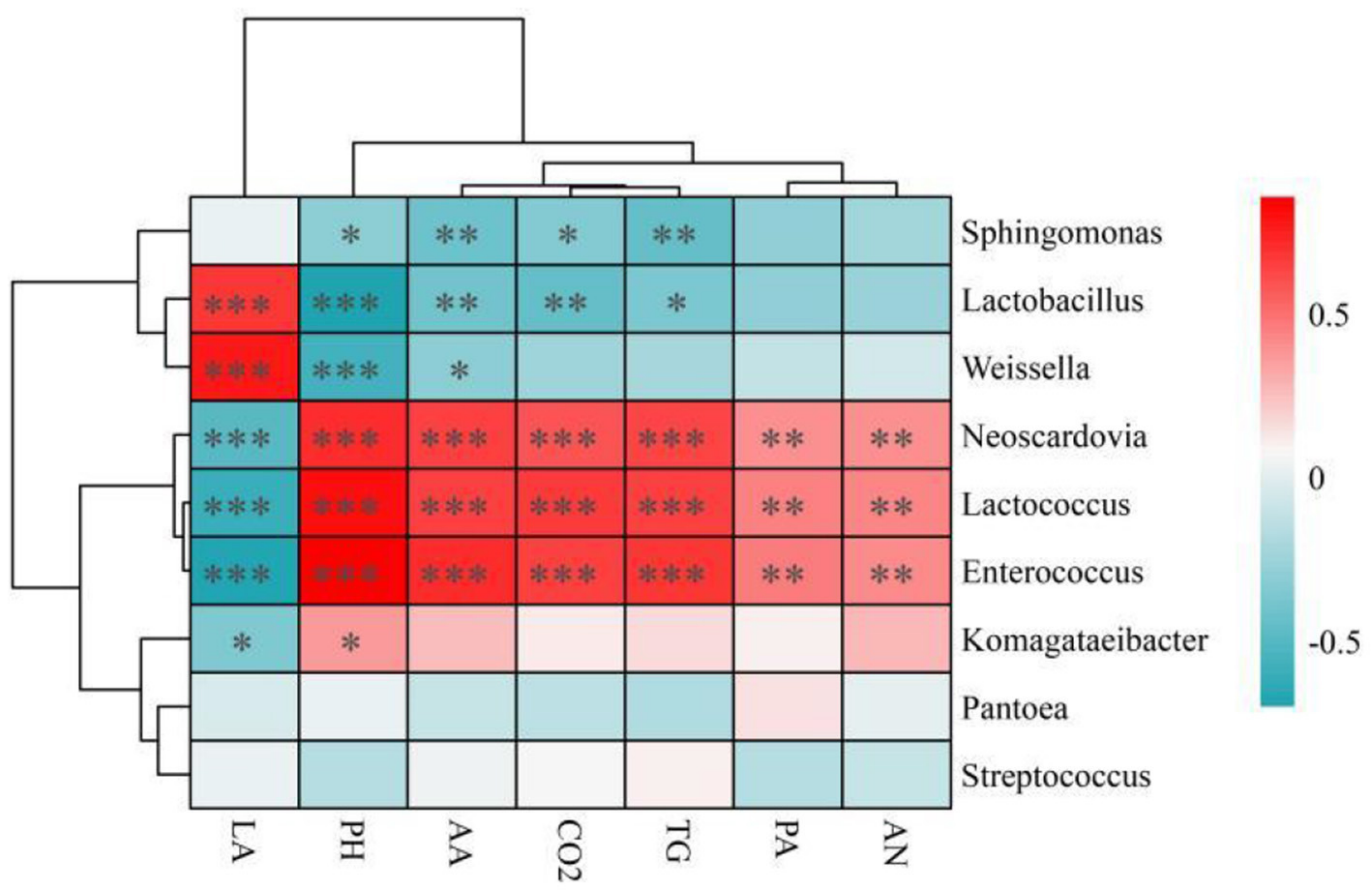
Pearson analysis between nutrient indices, fermentation indices and CO_2_ production and the bacterial community after ensiling. LA, lactic acid; AA, acetic acid; TG, total gas; PA, propionic acid; AN, ammonia nitrogen. This study utilized Pearson correlation coefficients for the heatmap analysis. Red squares indicate a positive correlation (values approaching 1) between taxa and production parameters, while blue squares represent a negative correlation (values approaching−1). Significance is denoted by *P < 0.05, **P < 0.01, and ***P < 0.001, with all *P*-values adjusted using the FDR method.

The results of the correlation analysis of the relative abundance of bacteria with fermentation products and gas production are shown in Figure 2. Regression analysis revealed that *Lactococcus* and *Enterococcus* were significantly positively correlated with pH, AA, PA, AN, and CO_2_ and significantly negatively correlated with LA; *Lactiplantibacillus* and *Weissella* were negatively correlated with pH, AA, PA, AN, and CO_2_ and significantly positively correlated with LA. Considering bacterial diversity (Figure 1) and gas production (Table 4), the results revealed an inextricable relationship between silage quality, CO_2_ production and silage bacterial community composition. Silage is a fermentation process driven by microorganisms, and in the presilage period, the process is carried out mainly by microorganisms such as *Lactococcus* and *Enterococcus*. A relatively high pH (>5) provides a suitable environment for the survival of microorganisms such as *Lactococcus* and *Enterococcus* (Ni et al., 2018; Andersen et al., 2005), and the presence of many *Lactococcus* and *Enterococcus* bacteria results in incomplete fermentation or poor fermentation quality, which explains the high CO_2_ production when the mixing ratios in CK treatment and B are high. *Lactiplantibacillus* and *Weissella* are the main microorganisms that produce lactic acid, and high levels of lactic acid are produced by *Lactiplantibacillus* and *Weissella* when they are present at relatively high relative abundances, decreasing pH and creating an acidic environment, which inhibits the activity of many aerobic microorganisms. This may be an important reason for the negative correlation between *Lactiplantibacillus* and *Weissella* and CO_2_ production. This finding is consistent with the results of Chen et al. (2021), who also reported that the relative abundances of *Lactococcus* and *Leuconostoc were* positively correlated with CO_2_ yield. Regression analysis revealed that *Neoscardovia* was significantly positively correlated with pH, AA, PA, AN and CO_2_ and significantly negatively correlated with LA. *Neoscardovia* is a specialized anaerobic bacterium in Bifidobacteriaceae in the Actinobacteria family that cannot tolerate strongly acidic environments (pH > 5; García-Aljaro et al., 2012; Mekadim et al., 2019), which may be one reason it was positively correlated with pH and CO_2_ production and negatively correlated with LA. Aerobic bacteria are the main microorganisms that produce CO_2_, and inhibiting the growth and reproduction of aerobic bacteria can effectively reduce CO_2_ production (Pahlow, 2003). *Komagatales* are gram-negative, specialized aerobic bacteria of the Acetobacteriaceae family that grow optimally at pH values ranging from 5.0 to 6.5, and they may contribute to the production of CO_2_ (Bishop et al., 2022). In addition, CO_2_ production may be related to some functions of bacteria. *Streptococcus* produces H_2_O_2_, which inhibits the growth of certain fungi in the phylum Ascomycetes, and these bacteria may contribute to the inhibition of aerobic bacterial growth. *Sphingomonas* may be useful in carbon sequestration and in reducing carbon dioxide emissions, and Wang et al. (2020) reported that rice fields with relatively high relative abundances of *Sphingomonas* presented relatively low CO_2_ emissions. The above results show that many microorganisms are involved in the fermentation of silage, which affect the quality of silage fermentation and CO_2_ production, and the bacterial community structure of silage can be changed by choosing an appropriate ratio of mixed silage substrates consisting of different types of forage materials. Appropriate additives further reduce the production of CO_2_ during ensiling.

## 4 Conclusions

In summary, silage nutrients are better balanced and silage fermentation is promoted when a mixed silage consisting of different forage crops is used, additives can further promote silage fermentation and improve silage quality, and the combined use of the two methods may be an effective measure to solve problems related to the low nutritive value of forage and poor silage fermentation. Mixing B and T can improve silage quality and reduce CO_2_ production during ensiling, and the best effect is achieved when the mixing ratio of B and T is 3:7. Additives can further improve silage quality when mixed B and T silage is used, and the addition of sour soup contributes the most to an improvement in fermentation quality and nutrient preservation, as evidenced by a relatively high LA content, low pH, low AN content and low CO_2_ production under sour soup treatment. Sour soup provides a sufficiently acidic environment for silage microorganisms and increases the relative abundance of *Lactiplantibacillus* and *Weissella*. A considerable amount of silage is produced in China annually, and the selection of suitable proportions of different forages for mixed silage and the addition of sour soup may be effective ways to improve silage quality and reduce greenhouse gas emissions during the ensiling process.

## Data Availability

The original contributions presented in the study are publicly available. This data can be found here: https://pan.baidu.com/s/1P7VkNLGhWuaGykGhmAWfZw with extraction code 1234.
